# Modulation of rotavirus severe gastroenteritis by the combination of probiotics and prebiotics

**DOI:** 10.1007/s00203-017-1400-3

**Published:** 2017-06-20

**Authors:** Guadalupe Gonzalez-Ochoa, Lilian K. Flores-Mendoza, Ramona Icedo-Garcia, Ricardo Gomez-Flores, Patricia Tamez-Guerra

**Affiliations:** 1 División de Ciencias e Ingeniería, Departamento de Ciencias Químico Biológicas y Agropecuarias, Universidad de Sonora, 85880 Navojoa, Sonora Mexico; 2 Facultad de Ciencias Biológicas, Departamento de Microbiología e Inmunología, Universidad Autónoma de Nuevo León, San Nicolás de los Garza, 66451 Nuevo León Mexico

**Keywords:** Rotavirus, Pathogenesis, Probiotics, Prebiotics

## Abstract

Annual mortality rates due to infectious diarrhea are about 2.2 million; children are the most vulnerable age group to severe gastroenteritis, representing group A rotaviruses as the main cause of disease. One of the main factors of rotavirus pathogenesis is the NSP4 protein, which has been characterized as a viral toxin involved in triggering several cellular responses leading to diarrhea. Furthermore, the rotavirus protein NSP1 has been associated with interferon production inhibition by inducing the degradation of interferon regulatory factors IRF3, IRF5, and IRF7. On the other hand, probiotics such as *Bifidobacterium* and *Lactobacillus* species in combination with prebiotics such as inulin, HMO, scGOS, lcFOS have been associated with improved generalized antiviral response and anti-rotavirus effect by the reduction of rotavirus infectivity and viral shedding, decreased expression of NSP4 and increased levels of specific anti-rotavirus IgAs. Moreover, these probiotics and prebiotics have been related to shorter duration and severity of rotavirus diarrhea, to the prevention of infection and reduced incidence of reinfections. In this review we will discuss in detail about the rotavirus pathogenesis and immunity, and how probiotics such as *Lactobacillus* and *Bifidobacterium* species in combination with prebiotics have been associated with the prevention or modulation of rotavirus severe gastroenteritis.

## Introduction

Severe diarrhea in the acute gastroenteritis is the primary cause of dehydration, which can lead to medical complications or death if left untreated (Hostetler et al. 2004). Annual mortality rates due to infectious diarrhea are about 2.2 million, and infants and very young children are the age group most vulnerable to severe gastroenteritis (Boschi-Pinto et al. 2008); children mortality were 578,000 worldwide (Liu et al. 2015). Viruses are the major agents of acute gastroenteritis in children up to 5-years-old (Chhabra et al. 2013). The most reported viruses associated with gastrointestinal infections are rotavirus (RV), norovirus, sapovirus, enteric adenovirus, and astrovirus (Elliott 2007). RV is the main cause of gastroenteritis in children; this virus is responsible for 453,000 deaths of children worldwide (Tate et al. 2012). The second place in the list of agents of acute viral gastroenteritis in children is for norovirus, which is related to 218,000 children deaths worldwide (Koo et al. 2010). Enteric adenovirus, sapovirus, and astrovirus have been detected in children up to 5-years-old with severe and mild gastroenteritis (Finkbeiner et al. 2009; Rezaei et al. 2012; Sdiri-Loulizi et al. 2011). Other viruses such as aichi virus, parechovirus, and bocavirus have been related to cases of acute diarrhea. Nevertheless, their participation as gastrointestinal pathogens remains unclear (Chhabra et al. 2013).

## Rotavirus

RV is a member of the genus *Rotavirus* within the family *Reoviridae;* mature viral particles are about 70–100 nm in diameter and possess a triple-layered icosahedral protein capsid composed of an outer layer, an intermediated layer, and an inner core layer. The RV genome contains 11 segments of double-stranded RNA (dsRNA), segments which encode six structural proteins (VP1–VP4, VP6, and VP7) and six non-structural proteins (NSP1–NSP5/NSP6) (Estes and Greenberg 2013). RV is classified in eight distinct groups (A to H), RVs A, B, and C are found in both humans and animals, whereas D, E, F, G, and H have been only found in animals (Matthijnssens et al. 2012).

RV causes significant diarrheal disease in infants and young of various mammalian and avian species (Estes and Greenberg 2013). Within RV, viruses are classified into serotypes and genotypes. The binary classification for RV is based on distinct types of the structural proteins in the external capsid VP7 (genotype G) and VP4 (genotype P). In 2008, a complete genome classification system was developed to RVA that assigns a specific genotype to each of the 11 genomic segments according to established nucleotide percent cutoff values (Matthijnssens et al. 2008). Most of the human RV associated to diarrheic disease worldwide are G1P[8], G2P[4], G3P[8], G4P[8], and G9P[8] with emerging genotypes such as G9 and G12 (Rahman et al. 2007; Santos and Hoshino 2005). These common human RVs may co-circulate within a single season which would be favorable for the formation of reassortant viruses and thereby to the genetic diversity of RV (Jain et al. 2014).

## Rotavirus pathogenesis

**Fig. 1:**
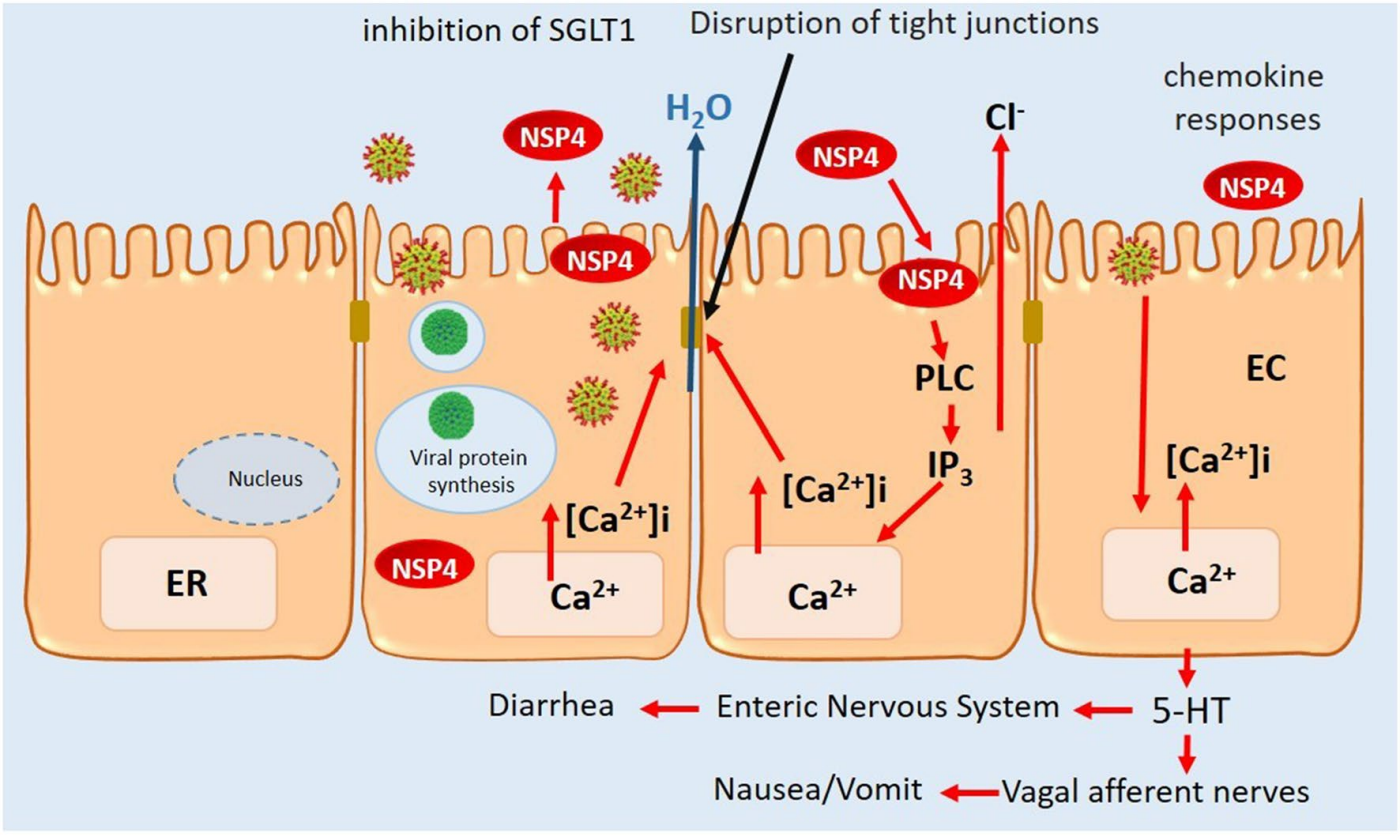
Model of RV infection and pathogenesis: virus entry, formation of viroplasms and replication, and release of virions and viral proteins such as NSP4; this protein mobilizes intracellular calcium from endoplasmic reticulum (ER). Released NSP4 affects uninfected cells, resulting in mobilization of intracellular calcium by a PLC-dependent pathway that activates chloride secretion. Tight junctions can also be disrupted by RV infection and by the NSP4 activity; the disruption of epithelial cell structure may lead to cell death and alterations of the paracellular pathway of fluid movement. RV infection activates epithelial cell signaling resulting in the activation of the enteric nervous system, intestinal secretion, and immune responses (Estes and Greenberg 2013; Ramig 2004)

RVs infection and replication are primarily in the nondividing, mature enterocytes near the tips of the small intestinal villi (Estes and Greenberg 2013). Nevertheless, RV infection may not be limited to the gut; recently, several cases of antigenemia and viremia have been reported, although the impact of systemic RV on disease burden remains to be determined (Blutt and Conner 2007; Estes and Greenberg 2013). The human RV pathogenesis is still unclear, some studies in volunteers, with animal models and recently in a novel in vitro human intestinal enteroids model (Saxena et al. 2016) point that the viral pathogenesis may be multifactorial and associated with several factors such as: (a) the viral infection to mature enterocytes in the lining of the gastrointestinal tract is related to enterocyte vacuolization and loss, crypt hyperplasia and villous blunting, which is associated with malabsorption by intestine; although, the presence of symptoms of such diarrhea has been reported before the epithelial damage is detected (Jourdan et al. 1997), (b) the activity of the RV non-structural protein NSP4 (Fig. 1), which has been characterized as a viral toxin inducing Ca^2+^-dependent Cl^−^ secretion associated with the inhibition of the Na^+^/glucose-cotransporter SGLT1, and alterations in cytoskeletal structure, in the integrity of the tight junctions and the regulation of Na^+^/K pump (Ball et al. 1996; Lundgren and Svensson 2001; Ousingsawat et al. 2011). This intracellular dysregulation in the enterocyte, together with the decreased expression of digestive enzymes, glucose malabsorption and activation of cystic fibrosis conductance regulator (CFTR)-independent Cl^−^ secretion, may be the cause of diarrhea (Ousingsawat et al. 2011), (c) the enteric nervous system is associated with RV secretory diarrhea and increased intestinal motility, the evidence of this association is the modulation effect of drugs that block this pathway in RV-induced diarrhea (Lundgren et al. 2000), (d) other factor in viral pathogenesis is the ability of RV to infect enterochromaffin cells (EC), as consequence serotonin (5-hydroxytryptamine) is released from EC and acts through the enteric nervous system inducing activation of vagal afferent nerves to brain structures associated with nausea and vomiting (Hagbom et al. 2011).

## Rotavirus immunity

The mechanisms responsible for generating protective immunity to RV infections and illness following natural infection are not completely understood, particularly in humans where it is difficult to study the acquired cellular immune response in young children due to limitations with timely and sufficient specimens (Estes and Greenberg 2013). Most of the knowledge about the immune response to RV has been studied in several animal models, but the most used are mice and pigs (Estes and Greenberg 2013). B or T cells knockout mice were observed to be chronically infected with RV; the same effect has been described in children with B or T cells immunodeficiency (Chhabra et al. 2013; Williams et al. 1998). CD4+ cells are critical for the establishment of protective long-term memory responses and important for the development of 90% of the RV-specific IgA (Kuklin et al. 2001). On the other hand, CD8+ T cells are associated with short-term protection against RV reinfection and with timely resolution of primary RV infection (Jiang et al. 2008). In the same animal model, intestinal tract homing of both B and T cells plays a major role in promoting RV immunity mediated by the integrin α_4_β_7_ and CCR9 (Jiang et al. 2008; Kuklin et al. 2001; Williams et al. 1998).

### Innate immune response and evasive strategies of rotavirus

In the absence of T cell help, a protective B cell response is present; nevertheless, this response is reduced compared with wild-type mice, and T cells can mediate their effect against RV infection in the absence of perforin, Fas, and interferon γ (Franco et al. 1997, 2006; Gilger et al. 1992). Apparently, T cells can clear infection more quickly and efficiently than B cells. CD8+ T cells can mediate primary RV infection and almost complete or partial protection from reinfection (Estes and Greenberg 2013).

**Fig. 2:**
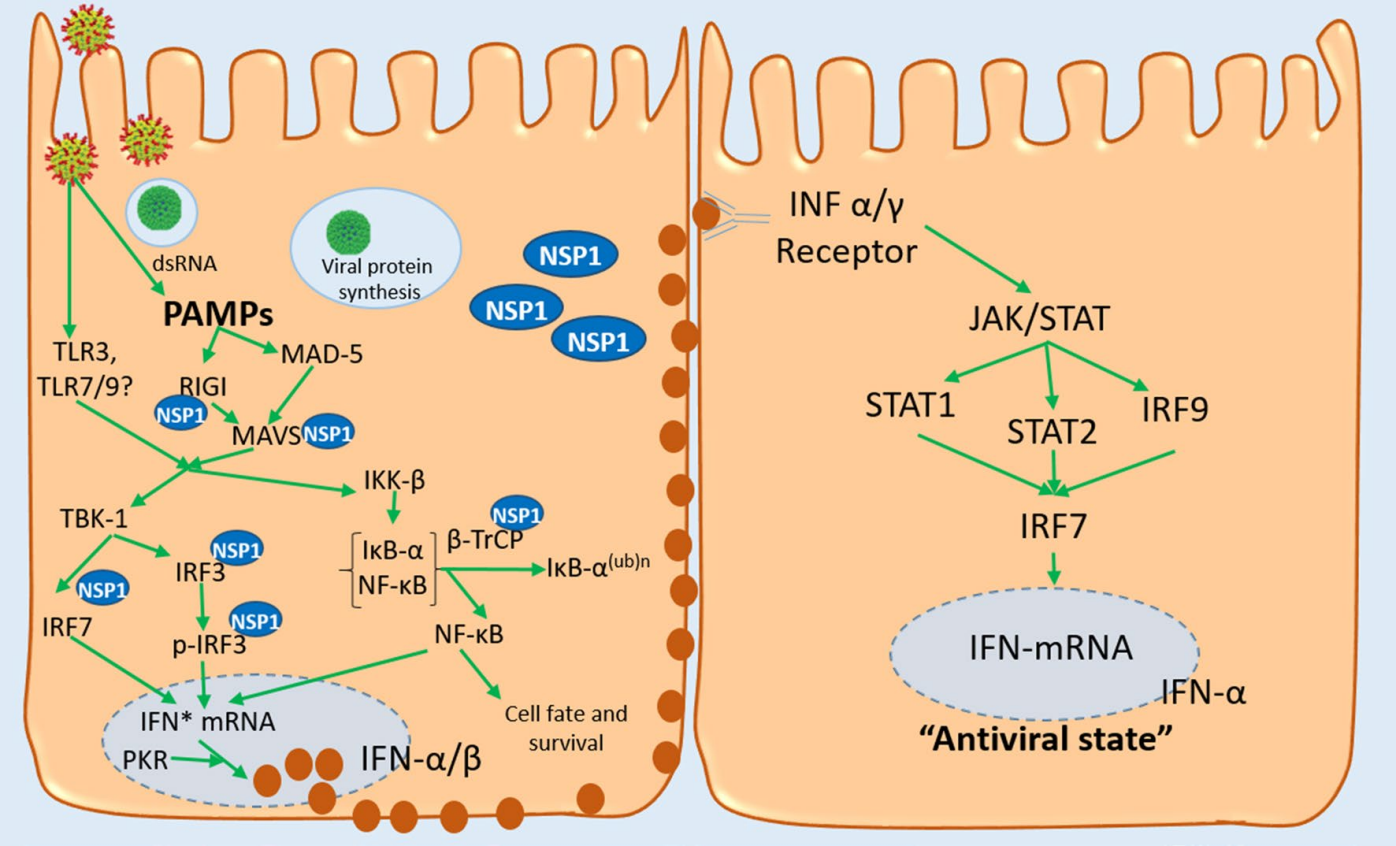
Rotavirus interactions with the host innate system: viral entry into cells and viral double strand RNA (dsRNA) induce the generation of pathogen-associated molecular pathways (PAMPs). As a result, cytosolic pathogen recognition receptors (PRRs), such as RIG-I and MDA-5 are activated, leading to mitochondrial-associated adaptor protein MAVS-dependent activation of transcription factor IRF3/IRF7. Activated IRF3/IRF7 translocates to nucleus, where it induces the transcription of several genes resulting in the transcription and expression of IFN-α/β. IFN secretion from rotavirus infected cells results in the establishment of antiviral state in bystander cells, mediated by signaling through the transcription factors STAT1, STAT2, and IRF9. The viral protein NSP1 induces proteosomal degradation of RIG-I, MAVS, IRF3, and IRF7. On the other hand, the induction of IFN in rotavirus infected cells also requires nuclear factor κβ (NF-κβ), following the proteosomal degradation of its inhibitory partner IκB-α; NSP1 can block this pathway by inducing the proteosomal degradation of β-TrCP, which is an essential co-factor for IκB-α degradation. As a result, NSP1 affects the quality and intensity of the interferon response (Arnold and Patton 2011; Estes and Greenberg 2013)

On the other hand, RV has developed multiple mechanisms to evade the innate immune response, particularly the interferon response (INF). The protein NSP1 has been characterized as an inhibitor of interferon (INF) production by inducing the degradation of interferon regulatory factor IRF 3, IRF5 and IRF7 in a host cell-dependent process (Fig. 2) (Arnold and Patton 2011). Due to the loss of IRF3, the expression of IFN-β is suppressed, the degradation of IRF5 is associated with the down-regulation of the activation of genes producing proinflammatory cytokines. Finally, the degradation of IRF7 is related to the decreased expression of type I IFN and to an altered activation of IFN-α genes (Barro and Patton 2007). NSP1 also mediate degradation of β-TrCP and inhibition of NFκB activation (Morelli et al. 2015). All these effects depend of the RV strain, and cell type, NSP1 from some animal RV degrade IRF3, IRF5, and IRF7; nevertheless, human RV NSP1 only degrades IRF5 and IRF7, which may result in less efficient inhibition of IFN response (Arnold and Patton 2011). NSP1 has also been associated with the degradation of other proteins such as the pattern recognition receptor (cytosolic receptor) known as retinoic acid-inducible gene I (RIG-I); TNF receptor-associated factor 2 (TRAF2), and the mitochondrial antiviral signaling protein (MAVS, also known as IPS-1, VISA, and Cardif). These data indicate that NSP1 can block innate immune signaling at both the transcriptional (IRF, NF-κB) and at pattern recognition receptor (PRR) level, but not signaling through the TLR3/TRIF pathway or PKR (Broquet et al. 2011). On the other hand, RV activates the PI3K/Akt pathway to prevent premature apoptosis, and it is also related to the post-transcriptional depletion of p53, possibly through the NSP1 activity; as a result, early cell apoptosis is prevented (Bagchi et al. 2010; Bhowmick et al. 2013).

## Probiotics and prebiotics vs rotavirus gastroenteritis

Current treatment of RV gastroenteritis consists of oral rehydration (oral rehydration solutions, ORS) to replace fluids and electrolytes lost by vomiting and diarrhea. Zinc supplementation improves the oral rehydration, and it is recommended by the WHO for children with acute gastroenteritis. Several other additives to the ORS formulation are currently under investigation; these include lactoferrin and lysozyme and various amino acids including glycine, alanine, and glutamine (Estes and Greenberg 2013). Additionally, the RV vaccines (Rotarix and RotaTeq) have shown to be safe and effective in the prevention of RV severe gastroenteritis. Nevertheless, they are not globally implemented due to their cost, storage and transport requirements (at 2–8 °C) and because of the lower protection offered in developing countries (Bines and Kirkwood 2015). Moreover, RV gastroenteritis seems to be modulated by nutritional interventions such as bioactive components of breast milk, probiotics or prebiotics (Rigo-Adrover et al. 2016).

Probiotics such as *Lactobacillus* and *Bifidobacterium* species, and *Saccharomyces boulardii* have been associated with the prevention of RV infection, to shorter duration and severity of RV diarrhea, to reduced incidence of reinfections and to the modulation of the immune response and viral shedding (Das et al. 2016; Lee et al. 2015; Maragkoudakis et al. 2010; Rigo-Adrover et al. 2017; Varyukhina et al. 2012).

**Fig. 3:**
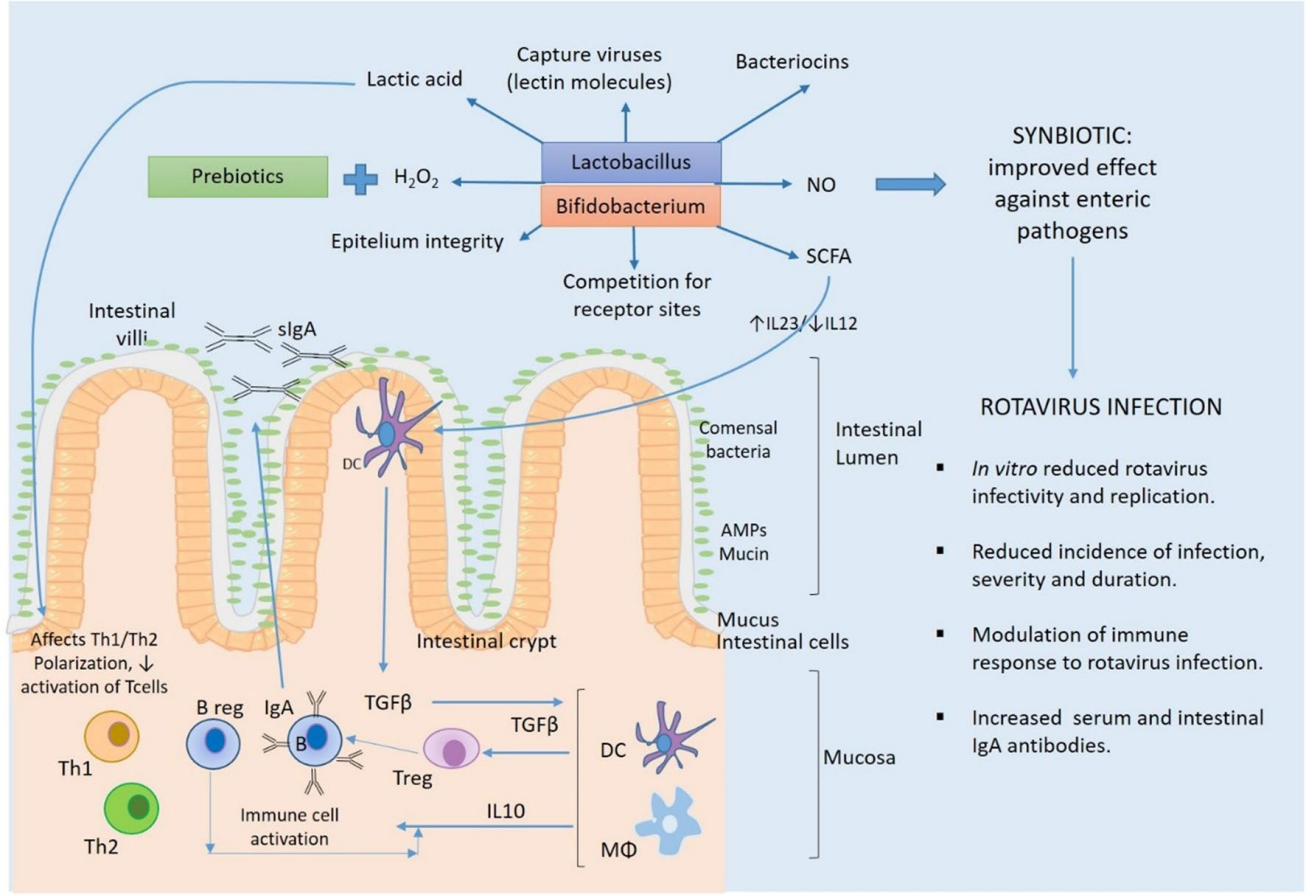
Prebiotics, probiotics, and gut immunity: interaction of prebiotics and probiotics such as *Lactobacillus* and *Bifidobacterium* species and the immune system, described from in vitro and in vivo assays with mice and gnotobiotic pigs. Prebiotics such as HMO, scGOS or lcFOS together with probiotics *Lactobacillus* and *Bifidobacterium* may improve the immune response against enteric pathogens. These probiotics inhibit some viruses by producing lactic acid, H_2_O_2_, NO, short-chain fatty acids (SCFA), bacteriocins, promotes and preserve the integrity of the epithelium, and compete with pathogens for intestinal epithelial cell (Vlasova et al. 2016)

Out of the reported probiotics showing potential as gut pathogens antagonists, some species of *Lactobacillus* and *Bifidobacterium* are commonly reported worldwide (Servin 2004). Focusing against RV, an in vivo evaluation on mouse demonstrated that oral administration of *Bifidobacterium breve* strongly protected against RV-induced diarrhea, thus observing an anti-RV IgA level increase in feces, mammary gland and intestine of treated mouse (Yasui et al. 1995). In other murine models, pathogen-free rats infected with SA11 RV strain and orally treated with *L. casei*, small intestine lesions, and RV infection level were reduced in all intestine sections, as well as diarrhea (Guérin-Danan et al. 2001). In vitro and in vivo studies revealed that some of the mechanisms of probiotics against RV infection are the production of antimicrobial substances (lactic acid, nitric oxide, H_2_O_2_ and bacteriocins), stimulation of antimicrobial peptides, mucin production by epithelial cells, stimulation of local adaptive (specific IgA response), and innate immune responses (Fig. 3) (Gänzle et al. 2000; Kaila et al. 1995). Moreover, *Lactobacillus* and *Bifidobacterium* species have been associated to the stimulation of production of cytokines IL25, IL33, TGF by intestinal cells; IL22, by innate immune cells; IL12, IL25, IL10 and TGF, by antigen-presenting cells; resulting in improved intestinal barrier function, reduced effector and increased regulatory immune responses (Vlasova et al. 2016).

On the other hand, prebiotics such as the sialic acid containing human milk oligosaccharides (HMO) has been associated to in vitro reduced RV infectivity and replication (Hester et al. 2013). HMO have also been associated with the reduction of the duration of RV diarrhea in piglets by modulating colonic microbiota and immune response to RV infection (Li et al. 2014). Moreover, a mixture of short-chain galactooligosaccharides (scGOS), long-chain fructooligosaccharides (lcFOS) and *Bifidobacterium breve* showed protection against RV infection in suckling rats (Rigo-Adrover et al. 2016). In children with acute RV gastroenteritis, the oral administration of a mixture of *Bifidobacterium lactis B94* and inulin as prebiotic showed a shorter duration of RV acute watery diarrhea (İşlek et al. 2014). On the other hand, a mixture of prebiotics such scGOS, lcFOS and pectin-derived acidic oligosaccharides mixture and heat-treated probiotics in fermented milk components in RV-induced diarrhea in suckling rats was associated with a decreased viral shedding and reduced clinical signs (Rigo-Adrover et al. 2017).

Although the probiotics and prebiotics mechanisms against RV are not well defined yet, there is some recent evidence about the beneficial effect of them in the viral pathogenesis and immune response modulation (Table 1). The activity of probiotics and prebiotics against RV pathogenesis may be attributable to decreased viral shedding possibly due to the interaction of probiotics (or their metabolites) and prebiotics with the viral particles avoiding the entry into enterocytes and as a consequence reducing the RV replication (Rigo-Adrover et al. 2017). Moreover, the in vitro effect of metabolites of *Lactobacillus casei*, and *Bifidobacterium adolescentis* was associated with a reduced expression of the RV enterotoxin NSP4 and reduced levels of Ca^2+^ liberation suggesting that cell will not reach the electrolyte imbalance caused by this pathway (Olaya Galán et al. 2016). On the other hand, the modulation of RV immune response by probiotics and prebiotics has been associated with a generalized antiviral response via pattern recognition receptor signaling and through promoting type I IFNs, which are key regulators of IFN signaling pathway (Ishizuka et al. 2016; Kang et al. 2015). *Bifidobacterium infantis* MCC12 and *Bifidobacterium breve* MCC1274 have been associated with a significant reduction of RVs titers in infected porcine intestinal epithelial cells (PIE); the beneficial effects of both bifidobacteria were associated with the reduction of A20 expression and improvements of IRF-3 activation, IFN-ß production, and MxA and RNase L expressions. The reduction of A20 is associated with the IFN stimulation response and IFN promoter dependent transcription by physically interacting with NF-κB-activating kinase/Traf family member-associated NFκB activator-binding kinase 1 and IKK-i/IKKe, and inhibiting dimerization of IRF-3 following engagement of TLR3 by dsRNA. In this regard, the up-regulation of MxA inhibits viruses by sequestering the newly synthesized viral proteins, and RNase L would be related to the lower RVs replication (Ishizuka et al. 2016). Thus, probiotics and prebiotics would be associated with generalized antiviral effect and to specific anti-RV activity.

**Table 1:** Effect of probiotics and prebiotics against rotavirus gastroenteritis

Probiotic species	Prebiotics	Assay description	Effect	References
*Lactobacillus casei* Shirota *Lactobacillus rhamnosus* GG	Not included	CLAB porcine cell line pre-incubated with selected LAB strains and then challenged with RV	Increased cell survival percentages, from 40% up to 80%	Maragkoudakis et al. (2010)
*Lactobacillus casei* *Bacillus thetaiotaomicron*	Not included	Human intestinal cultured cells HT29-MTX were incubated with bacteria-derived soluble factors and infected with RV	Decreased RV infection, more than 85% of HT29-MTX cells were not infected when *L. casei* spent culture supernatants were used Increased cell-surface glycan modification which was associated with a strong inhibition of RV entry	Varyukhina et al. (2012)
*Bifidobacterium longum* *Lactobacillus acidophilus*	Not included	In vitro antiviral activities of probiotic isolates on rotavirus Double-blind trial including children with viral gastroenteritis	Decreased antiviral activity by reduced plaque formation by 38 and 31% in Vero cells The duration of diarrhea was significantly shorter in the probiotic group	Lee et al. (2015)
*Saccharomyces boulardii*	Not included	Double-blind randomized controlled trial	RV-induced diarrhea was significantly shorter in the group with the probiotic	Das et al. (2016)
*Lactobacillus casei* *Bifidobacterium adolescentis*	Not included	In vitro assay in MA104 cells in a blocking RV model and intracellular model evaluating the NSP4 production and Ca^2+^ liberation measured by flow cytometry	Anti-RV effect in cells with metabolites of *Lactobacillus casei*, and *Bifidobacterium adolescentis* in the reduction of the NSP4 production and Ca^2+^ liberation	Olaya Galán et al. (2016)
Not included	Human milk oligosaccharides (HMO)	In vitro system for assessing cellular binding and viral infectivity/replication, and in a RV infection model in situ in piglets	Infectivity of RV was inhibited by sialylated HMO In situ assays with prebiotic + RV showed a lower viral replication, as assessed by enterotoxin NSP4 mRNA expression	Hester et al. (2013)
*Bifidobacterium lactis B94*	Inulin	Administration of probiotic and prebiotic in children with acute gastroenteritis.	The prebiotic-group shortened the duration of RV acute watery diarrhea	İşlek et al. (2014)
*Bifidobacterium breve*	Short-chain galactooligosaccharides/long-chain fructooligosaccharides	In vivo murine model	Reduced viral shedding Probiotic and prebiotic mixture enhanced the viral elimination and the host immune response against the virus	Rigo-Adrover et al. (2016)
*Bifidobacterium breve* *Streptococcus thermophilus*	Heat-treated (probiotic) fermented milk (FM) components Short-chain galactooligosaccharides./Long-chain fructooligosaccharides Pectin-derived acidic oligosaccharides	Rotavirus infection suckling rat model was used to evaluate improvements in the infectious process and in the immune response of supplemented animals	In the FM group: Reduced incidence, duration and severity of diarrhea and lower viral shedding Increased anti-RV antibodies in serum in the FM group; whereas in the prebiotics mixture group there was increased levels of intestinal anti-RV IgA	Rigo-Adrover et al. (2017)

## Conclusion

RV is the main cause of severe gastroenteritis in children up to 5-years-old worldwide. The current progress described in this review is the description of the strains of probiotics with the best effect against the RVs gastroenteritis, and how their effect may be improved by the presence of prebiotics such as inulin, HMO, scGOS, lcFOS, pectin-derived acidic oligosaccharides mixture and heat-treated probiotics in fermented milk components. Although more evidence is needed to support the beneficial effects and the mechanisms of prebiotics and probiotics against RV gastroenteritis severity; it is possible that the beneficial activity of probiotics and prebiotics are associated to: (a) the improvement in the intestinal microenvironment and the healthy intestinal microbiota balance strengthen the intestinal epithelial barrier, (b) the interaction of both probiotic (metabolites) or prebiotic with viral particles avoiding the RV cell entry, (c) increased generalized antiviral response, (d) decreased expression of the viral enterotoxin NSP4 and possibly of NSP1 and (e) the increased levels of specific anti-RVs IgAs. Together, all these factors would be associated to decreased RV infectivity, viral shedding, to shorter duration and severity of RV diarrhea, to the prevention of RV infection and reduced incidence of reinfections. Moreover, further studies are needed for the elucidation of the mechanisms of action of probiotics/prebiotics mixtures against RV severe gastroenteritis and the implementation of the effective and safe use of probiotic/prebiotics as preventive and therapeutic strategies in the management of RV gastroenteritis.
